# Effects of Feeding Different Diets on Carcass Performance and Mutton Quality of Horro Sheep

**DOI:** 10.1002/fsn3.70886

**Published:** 2025-08-29

**Authors:** Birmaduma Gadisa Muleta, Tesfaye Tadesa Desalegn, Abuye Tulu Demise

**Affiliations:** ^1^ Oromia Agricultural Research Institute Bako Agricultural Research Center Bako Ethiopia

**Keywords:** diet option, growth, Horro sheep, instrumental tenderness, meat quality

## Abstract

The study evaluated the carcass performances and meat quality of yearling Horro rams with an initial body weight of 25.35 ± 2.34 kg (mean ± SD) under different feeding regimes. The rams were grouped based on their initial body weight and tied in individual pens for 90 days. The experiment was arranged in a completely randomized block design with five treatments and five replications. All dietary treatments consisted of Rhodes grass hay offered ad libitum, supplemented with the following mixed diets: Treatment (T1) = 69% wheat bran (WB) + 29% noug seed cake (NSC) + 1% salt + 1% limestone, T2 = 56% wheat bran + 12% sorghum + 30% soybean + 1% salt + 1% limestone, T3 = 52% wheat bran + 14% maize + 32% NSC + 1% salt + 1 limestone, T4 = 47% wheat bran + 51% *Cajanas cajan* + 1% salt + 1% limestone, T5 = 47% wheat bran + 51% Dolichos lablab + 1% salt + 1% limestone. At the end of the experimental periods, rams were slaughtered to investigate carcass traits and meat quality. The results showed that the diets affected the carcass performances, edible and non‐edible offal components, and the meat quality of Horro lambs. However, there were comparable carcass components, total edible and non‐edible offal, and sensory and instrumental tenderness between T1, T2, T3, and T5 dietary treatments. In this study, most of the quality parameters did not show significant correlation with carcass yield parameters, except that Warner‐Bratzler Shear force (WBSF) had a significant relation (*p* < 0.05) with hot carcass and forequarter. Since the result exhibited comparable biological performance, the feeding strategy can be demonstrated depending on the availability of the diet to maximize the performance of the feedlot for Horro sheep. Future research is also warranted to explore the effects of this feeding regime on fatty acid composition, meat color, and chemical composition of Horro rams meat.

## Graphical abstract

Different feeding options affect Horro sheep meat quality. Horro sheep with a high‐quality diet can produce high‐quality meat. Dolichos lablab can be used as an alternative protein source for Horro sheep fattening.

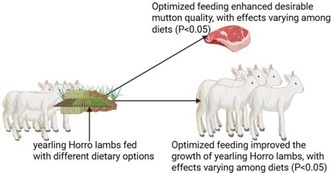

## 1 Introduction

Small ruminants contribute approximately 32.8% of the Ethiopian meat consumption (MOA 2024). Therefore, the small ruminants are receiving high priority for the red meat export market. Accordingly, small ruminant production has been prioritized in the Ethiopian Agricultural Transformation Plan (ATO 2020). The sheep population in Ethiopia is estimated at 42.9 million (Jemberu et al. 2022). It provides a source of protein and economic benefits, which help improve food security and reduce poverty in Ethiopia (Abebe et al. 2023). Producers target their lambs to attain the slaughter weight that the consumer desires quickly (Aschalew and Getachew 2013). However, Ayele and Urge (2019) reported that the mean carcass weight of Ethiopian sheep is less than 10 kg. Among various factors, inadequate nutrition plays a significant role in the lower growth performance, carcass weight, and carcass composition of sheep in Ethiopia (Ayele et al. 2018; Yami et al. 2018).

The Ethiopian government has given great attention to sheep breeding improvement to enhance weight gain. In contrast, meat consumers were satisfied with quality (Ayele and Urge 2019; Tefera et al. 2019) due to the increasing number of foreign visitors and high‐class hotels' demand (Yami et al. 2018). Among the quality indicators, eating quality, microbial quality, yields, chemical composition, traceability, and wholesomeness are the major ones (Joo et al. 2013; Muleta 2022).

For instance, meat produced in Ethiopia is not satisfactory for high‐end domestic hotels and competes with the international market (Birhanu et al. 2019; Ayza et al. 2025). Hence, Ethiopia has imported over 19 metric tonnes (MT) of meat per year from Italy, China, the United States, the Netherlands, the UAE, and South Africa to satisfy international hotels and supermarkets (Mummed and Webb 2015; Yami et al. 2018; FAO 2019). Supplementary feeding of sheep, with industrial by‐products, grain, hay, or silage, is necessary to meet the nutritional requirements (Ayele et al. 2018). However, the cost of feeds from agro‐industrial by‐products is too high. Hence, formulating diets from locally available crop grain as an alternative protein and energy source is important. Likewise, the inclusion of limestone (Atsbha et al. 2021), macro minerals (Ca and P), and trace minerals is important as components to improve meat quality (Patel et al. 2019).

Improving domestic meat is another area of government interest in the current scenario (ATO 2020). Horro sheep is one of the prolific sheep breeds in Ethiopia, which is produced in the mixed crop‐livestock production system of western Oromia for meat and income generation (Haile et al. 2011; MOA 2024). Different authors conducted different research activities for more than fourdecades on the breed and reported various results regarding slaughter body weight (20–32 kg), hot carcass weight (9–15 kg), and dressing percentage of slaughter weight (35%–48%), empty body weight (48%–53%) as compiled by Ayele and Urge (2019). Similarly, Yami et al. (2018) reported that the breed is ranked first for export and the high‐end domestic market in Ethiopia. However, information regarding meat quality traits in Ethiopian indigenous sheep, particularly for Horro sheep, is not well documented (Ayele and Urge 2019). Because the carcass quality of sheep is not well studied in the country, a few studies have focused on increasing meat volume. Hence, this experiment was designed to identify the best feeding ration, evaluate carcass yield, and meat quality traits of the Horro sheep breed fattened under different feeding regimes using locally available feed resources.

## 2 Materials and Methods

### 2.1 Study Area

Bako Agricultural Research Center (BARC) is located in Oromia Regional State, 258 km west of the capital, Addis Ababa, 8 km away from the nearest town, Bako, and 4 km from the highway road to Nekemte town, western Ethiopia. BARC lies between 9°6′ N latitude and 37°9′ E longitude at 1650 m above sea level (m a.s.l.). Agro‐ecology is mid‐altitude with high rainfall of 1238 mm year^−1^ and hot, humid weather, 13.3°C minimum and 28°C maximum annum^−1^.

### 2.2 Feed Preparation

The forage legumes hay of *Cajanus cajan* , Dolichos lablab, sorghum grain, maize grain, soybean grain, and Rhodes grass were produced in BARC in the main cropping season of June 2022. During the plantation, all prescribed agronomic practices were properly followed for both forage legumes, cereal, and soybean grain as recommended by Tulu et al. (2021), Balemi and Tufa (2019), and Dabessa (2023). During haymaking, both legume forages were harvested after they reached approximately 30%–40% flowering stage, field‐cured for 2–3 days depending on weather conditions, baled, and stored in a roofed hay barn (Tulu et al. 2024). The legume forages were then chopped into 3–5 cm lengths to make them more homogeneous for sampling and accessible to the animal using the *Maskinfabriken Taaruu Kerteminde, Dammark, 622* machine. The maize, sorghum, and soybean grains were harvested after 90% maturity and stored in a ventilated room for a month before starting the trial. A sufficient amount of wheat bran and noug cake required for the feeding periods was purchased from Guder town before the commencement of the experiment by calculating the amount of feeds as necessary for the whole feeding period. The *Bernhard Bruns Maschnefabrik Bad Zwischenahn hn I, 02 dB type M/B 15Z* machine was used to ground the grain and mix it with the concentrate diet.

### 2.3 Experimental Animals, Design, and Treatment Arrangement

Twenty yearling Horro sheep with an initial mean body weight of 25.35 ± 2.34 kg (mean ± standard deviation) were bought from the nearest local market. The animals were acclimatized to the new facilities and diets for 15 days with consideration for animal welfare. Deworming for internal parasites and vaccination for ovine pasteurellosis and sheep pox were conducted during quarantine periods. The rams were grouped according to their initial body weight and managed in individual pens for 90 days. The experiment was laid out in a complete randomized block design (RCBD) with five treatments and five replications. The animals were blocked by initial weight to one of the experimental treatments. The treatments (T) arrangement were: T1 = 69% wheat bran + 29% NSC + 1% salt + 1% limestone, T2 = 56% wheat bran + 12% sorghum + 30% soybean + 1% salt + 1% limestone, T3 = 52% wheat bran + 14% maize + 32% NSC + 1% salt + 1% limestone, T4 = 47% wheat bran + 51% *Cajanus cajan* + 1% salt + 1% limestone, T5 = 47% wheat bran + 51% Dolichos lablab + 1% salt + 1% limestone. Rhodes grass was provided ad libitum as a basal diet with weekly modification to account for 20% refusal, and free access to water was provided to all rams. The treatment diets were formulated on an iso‐nitrogenous and iso‐caloric basis to contain 20% crude protein (CP), as required for rams with a daily growth rate of 100 g, as recommended by the ARC (2007). Forage legume: Dolichos lablab and *Cajanus cajan* hay, grains; maize, sorghum, soybean, and a concentrate mixture (wheat bran and noug seed cake) were supplemented on an iso‐nitrogenous basis. A mixture of concentrate composed of noug seed cake and wheat bran was formulated in a proportion of 29%–69%, provided as a supplement to one of the treatment groups assigned as a positive control. Before the feeding trial commenced, the sample of supplement ingredients and Rhodes grass hay was analyzed for the chemical composition of DM content. Based on the chemical composition, the supplement rations were formulated and offered on a % DM basis.

### 2.4 Chemical Analysis

The chemical analysis of the experimental diets, refusals, and feces was carried out after taking representative samples and was conducted at the Holleta Agricultural Research Center nutrition lab, Ethiopia. Samples of feed offered, refusals, and feces were ground to pass a 1 mm sieve mesh. Standard methods (AOAC 2005) were followed to determine dry matter (DM), organic matter (OM), crude protein (CP), and ash contents. The CP content was estimated by the Kjeldahl method as *N* × 6.25. The content of neutral detergent fiber (NDF) and acid detergent fiber (ADF) was determined according to Van Soest et al. (1991), and acid detergent lignin (ADL) was determined according to Van Soest and Robertson.

### 2.5 Carcass Characteristics and Quality Evaluation

#### 2.5.1 Slaughtering Animals

After 90 days of feeding trials, all animals were fastened overnight, weighed, and slaughtered to evaluate slaughter weight, carcass, and non‐carcass yields, and palatability. After depriving feed and water overnight, animals were weighed using a spring balance of a capacity of 50 kg (NOPS). The hot carcass weight (HCW) was measured after the removal of internal organs, split into halves. The empty body weight (ESW) was calculated as slaughter weight (SW) minus gut contents.

### 2.6 Ethics Statement

All animal handling practices have followed the guidelines of treating animals, that is, on Animal Ethics and Welfare in Behavioral research and teaching, despite an established system for animal experiments (Guidelines for the Use of Animals 2012).

### 2.7 Sensory Quality Evaluation

#### 2.7.1 Panelist Screening

The pre‐selection of candidates was performed at Bako Agricultural Center based on their interest, availability, discriminative ability related to meat, familiarity with the product, and ability to express proportionality using scales accordingly. Following AMSA (2015) guidelines, panelists aged 20–40 years with no meat allergies were selected and trained.

#### 2.7.2 Panelist Training

The panelists who fulfilled the principal were presented for training. A 1‐day theoretical and a 1‐day panel session training were provided. The panelists scored each sample on a nine‐point hedonic scale for tenderness, flavor, and juiciness as follows: 9 = like extremely, 8 = like very much, 7 = like moderately, 6 = like slightly, 5 = neither like nor dislike, 4 = dislike slightly, 3 = dislike moderately, 2 = dislike, and 1 = dislike extremely. Each sample was labeled using a three‐digit code indicating treatment, panelists, and variables (AMSA 2015).

### 2.8 Steak Preparation and Presentation for Panelists

The steak preparation and cut was employed by one person to ensure uniformity (AMSA 2015). Cuts were browned in a charcoal‐heated oven. No ingredients were added to maintain the organic nature of the product. A similar procedure was followed to present the sample to panelists at similar room temperatures and in equal quantities. The room used for steak evaluation was far from the product preparation, noise, and aroma. The samples were served on clay pans on aluminum plates (white plates). Each assessor evaluated the three most important eating qualities (tenderness, juiciness, and flavor).

### 2.9 Determination of Water Holding Capacity (WHC)

The water‐holding capacity of meat was measured using the gravimetric method. A 50–100 g sample was weighed and placed in a plastic bottle for 24 h, then suspended. The fat part of the sample was removed and weighed before the hanging, then reweighed after being stored for 24 h (Honikel 1998; Wilborn et al. 2004). Water holding capacities (WHC) value was expressed, in percentage, from the total of the meat water composition (100%).

$$\mathrm{WHC}=\frac{\text{weight of sample}\;\mathrm{at}\;\text{first}-\text{weight after}\;24\;h}{\text{weight of initial}}\times 100$$

### 2.10 pH Value Determination

The meat pH evaluation is key for determining dry firm dark (DFD) and pale soft exudative (PSE). These PSE and DFD values are calculated from initial pH (after 45 min) and ultimate pH (after 24 h). We measured using a portable instrumental battery drive and a glass electrode digital pH meter. The pH meter probe was calibrated by inserting it into distilled water and a buffer solution, then touching the probe with meat and reading the pH value after about 30 s. DFD meat is typically defined as > 6.0; PSE meat is defined as < 5.4 (FAO 2001).

### 2.11 Instrumental Tenderness

The Warner‐Bratzler shear force (WBSF) apparatus was used to determine instrumental tenderness. The sample used for instrumental tenderness was cut from the *longissimus dorsi* between the 12th and 13th ribs. The sample used for evaluation was cooked and evaluated according to the AMSA (2015). The steak was cooked using pan broiling methods. After removing the steak from the cast iron skillet, it was allowed to cool down to room temperature for about an hour to evaluate instrumental tenderness using WBSF. After cooling, the steak was cut perpendicular to the muscle fiber direction, putting the knife tip on the heavy connective tissue side (dorsal) and the handle of the knife on the ventral side to expose the fiber direction. Six cores were removed parallel to the muscle fibers. The muscle fibers needed to run parallel with the core so that the shear was across the grain. The WBSF device was used to shear each core. The shear was across the middle (center) of each core. The peak values of WBSF were recorded in N (Newton) for each core, and average values for the six cores were used to present instrumental tenderness.

### 2.12 Data Analysis

The data on carcass yield, non‐carcass components, sensory traits, pH, WHC, and WSBF were analyzed using ANOVA based on the mixed model producers of SAS ver. 9.3. Dietary treatment was treated as a fixed effect, and individual animals were considered as a random effect. Normality and homogeneity of variance were assessed. The mean differences were compared using the least significant difference (LSD) at a 5% significance level.

The statistical model used was:

$$Y_{\mathrm{ij}}=\mu +T_{i}+B_{j}+E_{\mathrm{ij}}$$

where *Y*_ij_ is the dependent variable, *μ* is the overall mean, *T*_*i*_ is the effect of treatment, *B*_*j*_ is the block effect, and *E*_*ij*_ is the random error.

Correlation and regression analysis were employed to estimate the relation between quality indicators and to predict the hot‐harass weight.

## 3 Results and Discussions

The chemical compositions of the experimental feeds are presented in Table 1. The experimental feeds had comparable dry matter (DM%) and organic matter (OM%) contents. However, the compositions of ash, crude protein (CP), nutrient detergent fiber (NDF), acid detergent fiber (ADF), and acid detergent lignin (ADL) were different. Relatively high ash content was revealed in sorghum grain (9.9%), and the lower content was observed in wheat bran (3.82%). Noug seed cake (31.43%) and soybean grain (30.51%) contained the highest CP content, while maize grain (9.76%) had the lowest value. The NDF% content varied, ranging from 71.22% in pigeon pea hay to 30.06% in soybean grain. The ADF value ranged from 7.96% in wheat bran to 55.02% in sorghum grain. The composition of ADL observed in wheat bran and *Cajanas cajan* hay was 1.04% and 8.98%, respectively. The IVOMD contents varied, ranging from a greater content for wheat bran (75.32%) to a lower value for sorghum grain (44.88%). The mean value of fiber and non‐fiber chemical composition discovered in this study was consistent with previous reports (Tulu et al. 2018; Tulu et al. 2023; Gemechu et al. 2021; Kumsa et al. 2023). However, in the current study, the grass hay had the lowest CP (4.1%) and the highest fiber (75.9% NDF and 50.48% ADL) content.

**TABLE 1:** Chemical composition of experimental feeds and treatment diets.

Experimental diets	DM%	DM%						
		ASH	CP	OM	NDF	ADF	ADL	IVOMD
*Cajanas cajan* hay	93.06	6.39	21.98	93.61	71.22	51.84	8.98	58.77
Soybean grain	93.76	5.48	30.51	94.52	30.06	24.72	6.31	72.02
Maize grain	94.57	7.61	9.76	92.39	74.46	58.04	4.02	67.2
Dolicous lablab hay	94.26	8.44	22.19	91.56	58.6	45.81	6.37	65.44
Sorghum grain	94.14	9.9	9.74	90.1	64.07	55.02	5.35	44.88
Wheat bran	91.88	3.82	16.54	96.2	39.06	7.96	1.04	75.32
Noug cake	92.33	7.02	31.43	92.9	34.56	19.2	7.17	71.04
Rhodes grass hay	91.24	10.75	4.1	89.25	75.9	50.48	9.11	42.73

Abbreviations: ADF, acid detergent fiber; ADL, acid detergent lignin; CP, crude protein; DM, dry matter; IVOMD, in vitro organic dry matter; NDF, neutral detergent fiber; OM, organic matter; T, treatment.

### 3.1 Carcass Performance

The carcass performance of Horro rams managed under different dietary treatments is presented in Table 2. The overall slaughter weight (SW), empty body weight (EBW), and hot carcass weight (HCW) parameters were significantly different (*p* < 0.05) among dietary treatments. The measured slaughter weight was significantly lower in the T4 dietary group compared to the T1, T2, T3, and T5 diet groups. No significant difference (*p* > 0.05) was observed between T1, T2, T3, and T5 dietary groups on slaughter weight. The recorded empty body weight for animals assigned to dietary groups T2 (20.39 kg), T3 (20.67 kg), and T5 (21.89 kg) was comparable (*p* > 0.05) and higher than that of dietary groups T1 (19.68 kg) and T4 (18.07 kg). Regarding the hot carcass, animals assigned to dietary groups T2 (11.23 kg), T3 (11.34 kg), and T5 (12.08 kg) were comparable (*p* > 0.05) and higher than dietary groups T1 (10.4 kg) and T4 (9.42 kg). There was no significant variation (*p* > 0.05) between the T2, T3, and T5 dietary groups, and a similar result was observed for T1 and T4 (*p* > 0.05) in terms of empty body weight. The result obtained in the current study was consistent with earlier findings for some Ethiopian indigenous sheep breeds, such as Arsi‐Bale (Wegi et al. 2018; 22.85, 22.24, 10.89), Horro (Gemechu et al. 2021; 22.92, 20.41, 9.34), Washara (Gashu et al. 2017; 28.2, 23.5, 12.9), Blackhead (Kumsa et al. 2023; 22.12, 18.84, 11.59) for SW, EBW, and HCW, respectively. However, Atsbha et al. (2021) reported higher SW (37 kg), EBW (33.88 kg), and HCW (18.35 kg) for Begait sheep, whereas the current result of HCW (9.42–12.08 kg) was better than some previous reports for indigenous sheep breeds, such as Horro (Assefu 2012; 8–9 kg), Hararghe highland (Diriba et al. 2015; 6–8 kg).

**TABLE 2:** Slaughter performance of Horro sheep under different dietary supplementation.

Parameters	T1	T2	T3	T4	T5	SE	*p*
Slaughter weight (kg)	27.75^ab^	28.25^a^	28.13^a^	25.5^b^	29.63^a^	1.66	0.008
Empty body weight (kg)	19.68^bc^	20.39^ab^	20.67^ab^	18.07^c^	21.89^a^	1.19	0.0039
Hot carcass weight (kg)	10.4^bc^	11.23^ab^	11.34^ab^	9.42^c^	12.08^a^	0.70	0.0007
DPSBW	37.61^bc^	39.67^abc^	40.24^ab^	39.91^c^	40.73^a^	1.80	0.05
DPEBW	52.76^bc^	54.98^a^	54.75b^a^	52.15^c^	55.13^a^	1.37	0.008
Rib eye area (cm^2^)	18.56^ab^	19.63^ab^	22.13^ab^	17.31^b^	22.25^a^	0.32	0.023
Forequarter (kg)	5.69^b^	5.93^ab^	5.94^ab^	5.02^c^	6.43^a^	0.37	0.0013
Hindquarter (kg)	4.71^bc^	5.30^ab^	5.4^a^	4.40^c^	5.65^a^	0.41	0.0016
Front leg (kg)	2.29^bc^	2.48^ab^	2.47^ab^	2.11^c^	2.62^a^	0.15	0.0022
Hind leg (kg)	2.58^b^	2.73^ab^	2.95^a^	2.39^b^	2.98^a^	0.24	0.0104

*Note:* Different letters in the row indicated significant difference.

Abbreviations: DPEBW, dressing percentage of empty body weight; DPSBW, dressing percentage of slaughter body weight; T, treatment.

The slaughter weight, empty body weight, and dressing percentage results showed significant differences (*p* < 0.05) among dietary treatments. No significant differences (*p* > 0.05) were observed in DPSBW among T1, T2, and T4; however, T3 and T5 showed numerically higher values. The lower (*p* < 0.05) DPEBW values were recorded in diet groups of T1 and T4 compared to the other three treatments. This result indicated that the inclusion of sorghum, maize, soybean grain, and Dolicous lablab hay is important for rumen microbial and muscle development due to a balanced mix of energy and protein. The result of the current study was comparable with (Gashu et al. 2017; DPSBW 43.1–42.0 and DPEBW 53.2–54.0). However, the result was revealed higher than Gemechu et al. (2021) (DPSBW 33.94–39.36 and DPEBW 41.7–48.89). In contrast, the result was lower than Atsbha et al. (2021) (DPSBW 46.47–47.98, DPEBW 53.8–54.52) and Kumsa et al. (2023) (DPSBW 54.2–66.35, DPEBW 39.24–51.44). This variation in the final body weight, body weight change, and average daily weight gain might be due to the difference in the breed, type, and nutrient content of the dietary supplement, as well as the basal feed used for the respective studies. In general, the current study results of DPSBW and DPEBW relayed the average interval of indigenous Ethiopia sheep breeds as summarized by Ayele and Urge (2019) on DPSBW (41%–46%) and DPEBW (52%–58%) and DPEBW (52%–58%).

Rib eye area (REA) showed significant variation (*p* < 0.05) among treatments. The mean value of REA across treatments ranged from 22.25 to 17.311 cm^2^, the highest value obtained from T5 (22.25 cm^2^), and the lowest mean values were recorded from T4 (17.31 cm^2^). The REA indicates the proportion of muscle in the lean‐to‐bone ratio, which implies high retail yield. The current result was higher than previous reports of other Ethiopian indigenous sheep breeds (Wegi et al. 2018: 7.3–8; Worku et al. 2020: 9.2–9.9, 6.4–9.93; Atsbha et al. 2021: 13–16.13; Kumsa et al. 2023: 7.61–11.45). This result indicated that Horro rams can produce more back muscle than other indigenous sheep breeds in Ethiopia. Fore and hind quarter and front and hind leg carcass yield showed significant (*p* < 0.05) differences among dietary treatments. The highest fore and hind quarter, front and hind leg carcass yields were obtained from T5, yielding 6.43 kg (fore quarter), 5.02 kg (hind quarter), 2.62 kg (front leg), and 2.98 kg (hind leg). The lowest (*p* < 0.05) fore and hind quarter, front leg values were recorded in the diet group of T4 compared to the other three treatments. The mean value of fore and hind quarter, front and hind leg carcass yields was recorded from T4: 5.02, 4.40, 2.11, and 2.39 kg, respectively. The result revealed that the inclusion of Dolochos lablab in Horro rams fattening formulation enhanced muscle development more than pigeon pea, noug cake, and soybean grain. The current result was supported by the earlier finding of the inclusion of Dolochos lablab in Horro sheep fattening as hay (Tulu et al. 2024) and silage (Gemechu et al. 2021), and Horro ewes' reproductive performance (Tulu et al. 2023), Blackhead (Kumsa et al. 2023), and Begait (Berhane 2022) for sheep fattening as hay.

### 3.2 Edible and Non‐Edible Components

Edible and non‐edible components of Horro rams supplemented under different dietary treatments are present in Table 3. The offal components were categorized into edible (kidney, liver, heart, empty gut, reticulum‐rumen, omasum, abomasum, intestine, tail, tongue, and head) and non‐edible (blood, spleen, lung with trachea, abdominal fat, testicle, gut content, skin, and feet) based on local dietary customs. The mean value of kidney, abdominal fat, empty gut, reticulum–rumen, head, and skin showed significant variation (*p* < 0.05) among dietary treatments (Table 3). The significant variation (*p* < 0.05) was revealed between dietary groups of T1, T2, T3, and T4 on the head. Similarly, Gemechu et al. (2021) reported that the kidney, skin, and head showed a significant (*p* < 0.05) difference for the Horro sheep feed mixture of elephant grass and Dolichos lablab silage, and Adnew et al. (2021) for Farta sheep. This result is supported by Riley et al. (1989), who reported that the difference in the internal organs is more influenced by the age, breed, and sex of the animals rather than the plane of nutrition, which is consistent with the current result and early findings. Blood, lungs with the trachea, liver, heart, spleen, testicle, omasum–abomasum, intestine, tail, tongue, and total non‐edible organs did not show a significant difference (*p* > 0.05) in dietary treatment. The lack of significant variation among treatment diets on internal organs might be due to the low level of anti‐nutritional compounds in the formulated diet. Similarly, Gashu et al. (2017) reported that blood, testicles, full gut, skin, and total non‐edible organs did not show significant variation (*p* > 0.05) for intact and castrated Washara sheep fed high and low dry matter levels. Ahamefule et al. (2006) reported that increased weights of internal organs are commonly used as evidence of toxicity or the presence of antinutritional factors in the diet. The higher mean value of empty gut (2.04 kg), reticulum–rumen (590 g), head (1.7 kg), and skin (2.82 kg) was recorded from rams that received T5. In general, the weight of total edible (TEO) and non‐edible organs (TNEO) was not affected by treatment in the current study. The lack of significance of TEO and total TNEO was reported in many scientific studies, Faji et al. (2019), Adnew et al. (2021) and Tulu et al. (2023). This potency is due to tissues not easily affected by the normal nutritional status and growth rate of the animal, rather than the availability of toxicity and antinutritional factors in formulated diets. The current result of TEO is comparable with Gemechu et al. (2021) (5.9) and Gashu et al. (2017) (5.28), while lower than Wegi et al. (2018) (9.6) and Kumsa et al. (2023) (7.8). The lack of significant difference in the full gut among different diets received by sheep in the current study might be due to the fasting periods before slaughtering being equal.

**TABLE 3:** Edible and non‐edible non‐carcass components of Horro sheep under different dietary supplementation.

Parameters	T1	T2	T3	T4	T5	SE	*p*
Tongue (g)	100	100	120	110	120	0.02	*0.42*
Head (kg)	1.43^b^	1.49^b^	1.44^b^	1.36^b^	1.7^a^	0.12	*0.01*
Reticulum–rumen (g)	590	570	570	530	590	0.06	***0.03***
Omasum (g)	80	90	60	90	90	0.02	*0.55*
Abomasum (g)	180	170	120	130	170	0.05	*0.61*
Intestine (g)	1140	1030	1080	900	1110	0.12	*0.18*
Tail (g)	520	660	480	290	0.56	0.21	*0.39*
Liver (g)	450	420	410	440	480	0.04	*0.34*
Heart (g)	140	130	140	120	130	0.19	*0.62*
Kidney (g)	130	120	130	110	150	0.02	*0.05*
Empty gut (kg)	1.98	1.86	1.83	1.61	2.04	0.18	*0.05*
TEO (kg)	**5.25**	**5.41**	**5.01**	**4.31**	**5.72**	**0.55**	***0.0740***
Spleen (g)	60	60	70	60	70	0.01	*0.28*
Testicle (g)	410	350	380	400	370	0.05	*0.32*
Abdominal fat (g)	90	120	110	80	100	0.05	*0.042*
Full gut (kg)	8.08	7.87	7.46	7.43	7.73	1.28	*0.63*
Blood (g)	958.8	828.8	885.7	803.8	777.5	0.17	*0.67*
Lung + trachea (g)	460	460	420	400	460	0.04	*0.26*
Skin (kg)	1.53^ab^	2.71^b^	2.70^a^	2.31^b^	2.82^a^	0.25	*0.013*
Feet with hoof (g)	670^b^	730^a^	680^ab^	650^b^	730^a^	0.04	*0.02*
TNEO (kg)	**13.23**	**13.1**	**12.70**	**12.07**	**13.05**	**13.86**	***0.23***

*Note:* Different letters in the row indicated significant difference.

Abbreviations: SE, standard error; T, treatment; TEO, total edible organ; TNEO, total non‐edible organ.

### 3.3 Meat Quality Characterization

The results of pH, cooking loss, drip loss, sensory evaluation, and instrumental tenderness are presented in Table 4. There were no significant differences (*p* > 0.05) in quality parameters considered in the current study, except for juiciness and WBSF among treatments. Meat pH was not affected by diet (*p* > 0.05). The initial pH (ipH) values (6.5–6.63) and ultimate pH (upH) (5.63–5.7) were within the normal ranges of the import–export market (Adzitey and Nurul 2011; Ponnampalam et al. 2017). Therefore, the current study showed that animals were in good physical condition with sufficient glycogen reserves in the muscle to build an optimum level of lactic acid production. Indeed, the result was complemented by Merera et al. (2013) and contradicted by Abebe et al. (2010), Legese and Fadiga (2014), and Yami et al. (2018) regarding the cases of meat from the highland Ethiopian sheep breeds, which quickly darken and have a short shelf‐life.

**TABLE 4:** Meat quality characteristics of Horro sheep under different dietary supplementation.

Parameters	T1	T2	T3	T4	T5	SE	*p*
Initial pH (ipH)	6.5	6.63	6.5	6.63	6.55	0.12	0.61
Ultimate pH (upH)	5.63	5.7	5.65	5.68	5.7	0.14	0.94
Cooking loss (%)	25.95	27.57	27.17	28.64	23.27	7.15	0.85
Time of cooking (min)	5	4.75	6.75	4.25	4.75	1.39	0.23
Drip loss (%)	3.57	10.52	17.61	12.25	11.46	9.01	0.33
Flavor	8	7.80	8.23	7.23	7.86	0.39	0.38
Juiciness	7.73^b^	7.50^b^	7.78^ab^	7.66^b^	8.11^a^	0.23	0.033
Tenderness	7.82	7.41	7.48	7.84	7.94	0.41	0.20
WBSF (N)	24.20^ab^	22.32^ab^	25.95^ab^	34.13^a^	17.84^b^	9.24	0.044

*Note:* Different letters in the row indicated significant difference.

Abbreviations: DFD, dark firm dry; PSE, pale soft exudative; SE, standard error; T, treatment; WBSF (N), Warner Brazteler shear force in Newton.

Cooking loss, time of cooking, and drip loss were not affected by treatment diet (*p* > 0.05). The mean value of cooking loss in the current study ranged from the lowest in T5 (23.27%) to the highest in T4 (28.64%). Even though cooking loss was affected by treatment in the report of Atsbha et al. (2021) for Begait sheep (24.18%–26.12%), the result was comparable to the current result. TuluTulu et al. (2023) reported that the cooking loss ranged from 26.7% to 33.9% for Hararghe highland sheep, and Chulayo and Muchenje (2013) reported 35.5% for Blackhead Persian and 33.0% for Dorper sheep. The variation among findings might be due to cooking type, post‐mortem meat handling, temperature used during cooking, meat aging, and dietary treatment. In general, the cooking loss values of the current study were in an acceptable range (14%–41%), which was consistent with findings by Ayele et al. (2018), Atsbha et al. (2021), Worku et al. (2020), and Tulu et al. (2023). The time to cook meat rams fed different dietary treatments ranged from the lowest T4 (4.25 min) to the highest in T3 (6.75 min). The highest percentage of drip loss was recorded from T3 (17.61%) and the lowest from T1 (3.35%). The current result of drip loss was higher than Tulu et al. (2023), who reported drip loss in the range of 4.4 to 2.1 for Hararghe highland sheep breed. Meat water retention indicators such as drip loss (DL) and cooking loss (CL) are linked to the capacity of the meat muscle to retain water (Corazzin et al. 2019), which helps to determine the quality measure because it affects juiciness and tenderness (Lima et al. 2018 ).

In the current study, sensory tenderness and flavor were not affected by dietary treatments. The mean value of flavor ranged from the lower T4 (7.23) to the higher T3 (8.23), despite sensory tenderness ranging from the lower T2 (7.41) to the higher T5 (7.94). The result was higher than Ayele et al. (2018), who reported flavor in the range of 6.5–6.9 and tenderness in the range of 6.5–7.2 from three Ethiopian indigenous sheep breeds treated under different concentration levels. This might be due to the inclusion of calcium in the diet, which has improved tenderness by activating the natural endogenous calpain proteinases (Bhat et al. 2018). There were significant differences (*p* < 0.05) in juiciness and instrumental tenderness among dietary treatments. The mean value of juiciness ranged from the lower T1 (7.73) to the higher T5 (8.11). Instrumental tenderness was affected (*p* < 0.05) by diet. The mean value of WBSF ranged from 17.84 to 34.13 N. The comparable WBSF result was reported by Worku et al. (2020), 27.96–31.34 N for Gumuze, Rutna, and Wasgara sheep breeds of Ethiopia, and Chulayo and Muchenje (2013) for Blackhead, Dorper, and SAMM sheep breeds with mean values of 22.9, 26.4, and 26.8 N, respectively, in South Africa. Whereas Atsbha et al. (2021) reported higher mean values of 40.43–42.67 N for Begait and Tulu et al. (2023) lower mean values of 10.79–18.63 N for Hararghe highland sheep, Ethiopia. This result designates that the variation in instrumental tenderness (WBSF) among Ethiopian sheep requires investigation into the causes of variation, which might be due to dietary treatment or genetic makeup.

### 3.4 Carcass Yield and Sensory Qualities Correlation

The Pearson correlation of carcass yield and sensory quality is presented in Table 5. Fore quarter (FQ) is strongly (*p* < 0.0001) correlated with hindquarter (HQ) (*r* = 0.9); similarly, hot carcass (HC) was strongly correlated (*p* < 0.0001) with HQ (*r* = 0.98) and FQ (*r* = 0.97). The result of sensory tenderness showed a moderate (*p* < 0.001) correlation with juiciness (*r* = 0.64). WBSF was moderately correlated (*p* < 0.05) with FQ (*r* = −0.53) and weakly with HC (*r* = −0.40). The rib eye area is moderately correlated (*p* < 0.001) with HQ (*r* = 0.73), FQ (*r* = 0.6), and HC (*r* = 0.68). The result of initial pH, ultimate pH, and flavor did not indicate a significant relation (*p* > 0.05) with other parameters. However, Chulayo and Muchenje (2013) reported that ultimate pH was highly correlated with meat tenderness, color, and WHC. The WBSF values were correlated with carcass weight. REA was correlated with carcass weight and sensory tenderness. This result was expected due to the high carcass consequence of high muscle development, which can indicate high surface area. Regarding correlation with sensory tenderness, animals in good body condition can be expected to produce good marbling, which is highly influenced by meat tenderness.

**TABLE 5:** Pearson correlation between carcass yield and sensory quality.

	HQ	FQ	HC	ipH	upH	Flv	Jiuce	Tend	WBSF	REA
HQ	1									
FQ	0.9***	1								
HC	0.98***	0.97***	1							
ipH	0.23^ns^	0.17^ns^	0.2^ns^	1						
upH	−0.04^ns^	0.12^ns^	0.04^ns^	0.23^ns^	1					
Flv	0.12^ns^	0.12^ns^	0.12^ns^	0.01^ns^	0.09^ns^	1				
Juice	0.10^ns^	0.18^ns^	0.14^ns^	−0.23^ns^	0.08^ns^	0.25^ns^	1			
Tender	−0.32^ns^	−0.24^ns^	−0.29^ns^	−0.11^ns^	−0.21^ns^	0.4^ns^	0.64**	1		
WBSF	−0.33^ns^	−0.53*	−0.40*	0.15^ns^	−0.29^ns^	0.27^ns^	−0.05^ns^	0.37^ns^	1	
REA	0.73**	0.6**	0.68**	0.25^ns^	−0.1^ns^	−0.1^ns^	0.004^ns^	−0.43*	−0.16^ns^	1

*Note:* * singificant at 0.05, ** significant at 0.01, *** significant at 0.0001.

Abbreviations: Flv, flavor; FQ, forequarter; HC, hot carcass; HQ, hindquarter; ipH, initial pH; REA, rib eye area; Tend, tenderness; upH, ultimate pH; WBSF, Warner Bratzlar shear force.

### 3.5 Prediction of Carcass Yield From SBW, EBW, REA, TNEO, and TEO

The prediction of hot carcass yield from slaughter, empty body weight, and total edible and nonedible organs is presented in Table 6. Meat yield can be effectively predicted from slaughter weight with a coefficient of determination (*R* ^2^) of 0.78. At 0.97 of coefficient of determination, hot carcass yield was predicted well from empty body weight. REA, TEO, EBW plus REA, and SBW plus REA also predicted Horro sheep mutton carcass yield with a coefficient determination of 0.68, 0.74, 0.97, and 0.89, respectively. This indicated that the slaughter body weight, empty body weight, REA, and TEO could be reliable measurements in the estimation of carcass yield, whereas TNEO was less effective in predicting lower hot carcass yield.

**TABLE 6:** Prediction of hot carcass from SBW, EBW, REA, total no‐carcass (TEO and TNEO).

Equations	*R* ^2^	*p*	Actual value	Estimation value
**MY = 0.47SBW − 2.11**	0.78	0.0001	10.89	10.9795
MY = 0.47EBW − 2.67	0.97	2.62E‐12	10.89	8.7134
MY = 0.21REA + 6.66	0.68	0.0001	10.89	10.8558
MY = 0.62EBW + 0.034REA − 2.35	0.97	1.38E‐11	10.89	13.34572
MY = 0.41SBW + 0.05REA − 1.5	0.89	0.0001	10.89	10.9173
MY = 1.49TEO + 3.23	0.74	0.0002	10.89	10.8886
MY = 0.44TNEO + 5.2	0.49	0.028	10.89	10.8452
MY = 0.1TNEO + 1.36TEO + 2.58	0.75	0.0002	10.89	10.8534

Abbreviations: EBW, empty body weight; MY, meat yield; REA, rib eye area; SBW, slaughter body weight; TEO, total edible organs; TNEO, total non‐edible organs.

## 4 Conclusions and Recommendations

In the current study, slaughter weight (SW), empty body weight (EBW), and hot carcass weight (HCW) were affected by dietary treatments. However, pH, cooking loss, drip loss, flavor, and sensory tenderness were not affected by diet, while juiciness and instrumental tenderness were affected by the treatment option. The diet options of T1, T2, T3, and T5 exhibited comparable rib eye area, total edible and non‐edible carcass, sensory tenderness, and meat pH. The Pearson correlation result of carcass yield with palatability and instrumental tenderness showed non‐significant correlations. Thus, depending on their availability, supplementation of lambs' inclusions of grain like maize, sorghum, soybean, and Dolochus lablab hay (T2, T3, and T5) crops was comparable with the concentrate mixture. This result indicated a great potential of alternative grain‐legume‐based crops as a viable substitution for conventional concentrate mixture in Horro sheep fattening. To validate the viability of the findings and effectively utilize the current findings, an on‐farm experiment under the typical farmer‐managed conditions is essential. In the future, determining the optimum inclusion of forage legumes and cereal and pulse crops grain in sheep diet, the effects of feeding regime on fatty acid composition and technological meat qualities such as color and lean‐to‐fat ratio are crucial.

## Author Contributions

**Birmaduma Gadisa Muleta:** conceptualization ideas, writing – original draft, review and editing. **Tesfaye Tadesa Desalegn:** collecting data – supervising the experiment, review and editing. **Abuye Tulu Demise:** collecting data, reviewing, and editing the manuscript.

## Conflicts of Interest

The authors declare no conflicts of interest.

## Data Availability

The data used to support the findings of this study are available from the corresponding author upon request.
